# Endoscopic resection for verrucous venous malformation and capillary arteriovenous malformation: a deep approach to superficial lesions

**DOI:** 10.3389/fsurg.2025.1515564

**Published:** 2025-03-25

**Authors:** Zhengtuan Guo, Chong Xie, Weilong Lin, Peihua Wang, Weijia Yang, Huaijie Wang

**Affiliations:** Department of Pediatric Surgery and Vascular Anomalies, Xi’an International Medical Center Hospital, Xi’an, China

**Keywords:** surgery, endoscopy, venous malformation, vascular malformation, capillary arteriovenous malformation, verrucous venous malformation

## Abstract

**Background:**

Open surgery is the treatment of choice for verrucous venous malformation (VVM) and capillary arteriovenous malformation (CAVM) with overgrowth. The study aimed to report an innovative endoscopic resection technique for subcutaneous vascular malformations with superficial lesions at our center.

**Methods:**

We retrospectively reviewed the medical records of patients who underwent endoscopic resection for VVM or CAVM between September 2019 and July 2024.

**Results:**

The current cohort includes 14 female and 4 male patients, with ages ranging from 1 year to 8 years. Diagnoses included classic VVM (*n* = 10), VVM—subcutaneous variant (*n* = 4), and CAVM (*n* = 4). Endoscopic surgery uses two or more small ports in a gas-inflated manner. Surgery included radical resection, partial resection of the subcutaneous mass, and debulking of adipose overgrowth. Technical success was achieved in all patients. Local skin necrosis (area <1 cm^2^) occurred in two patients. No recurrence was observed during follow-up.

**Conclusion:**

Endoscopic resection is a minimally invasive, feasible, and safe technique for VVMs in selected CAVM. Better cosmetic results can be expected in patients with superficial lesions using this endoscopic surgical technique.

## 1 Introduction

**Figure 1:**
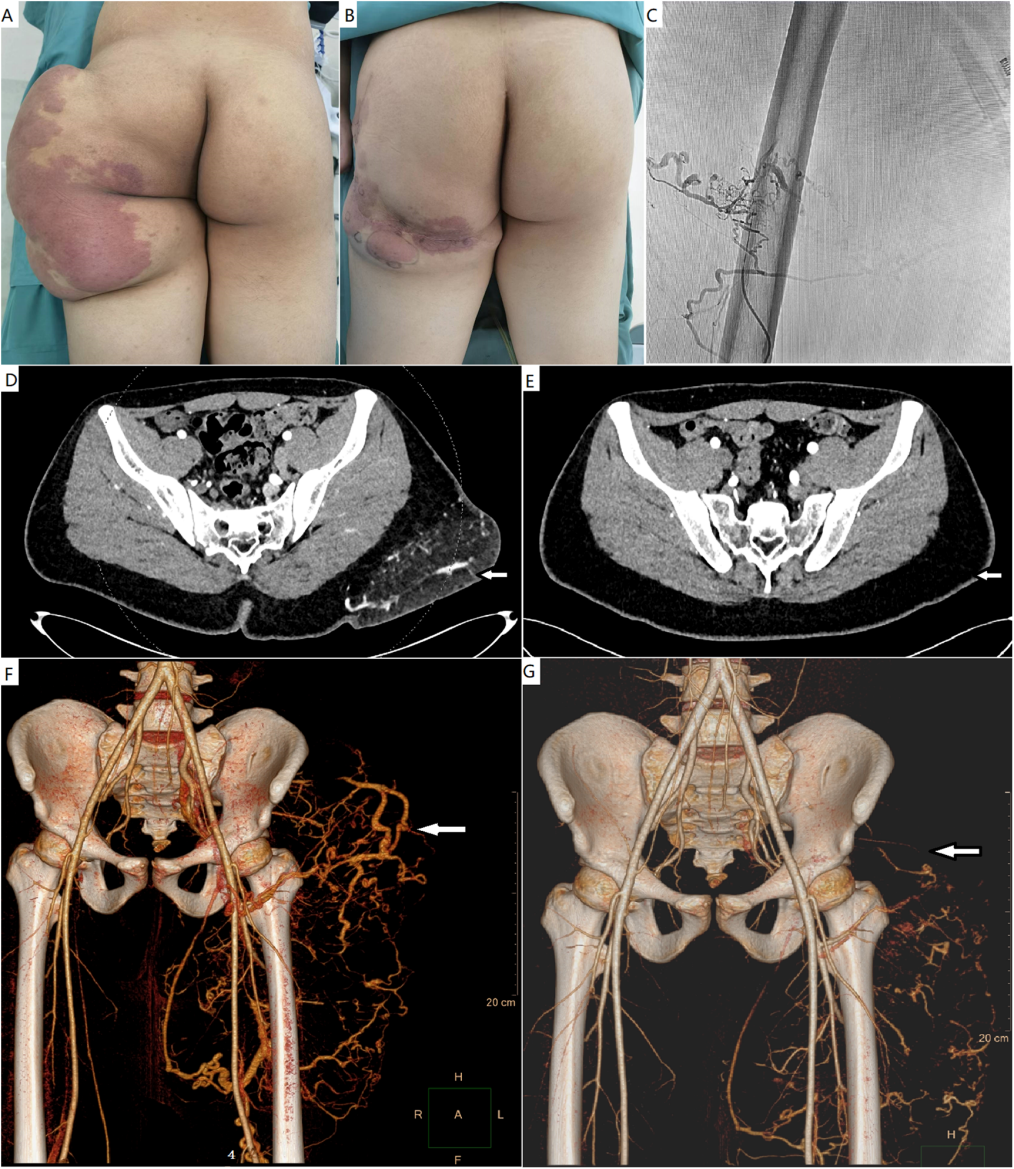
**(A)** A male patient with a CAVM with massive fatty overgrowth in the left buttock. **(B)** He underwent debulking surgery. **(C)** One year after surgery, ethanol embolization was performed to ablate the residual CAVM. **(D,E)** Pre- and post-procedural computed tomography studies with contrast enhancement. The mass with AVM was successfully debulked. **(F,G)** Pre- and post-procedural computed tomography angiography showing the markedly ablated vascular component. CAVM, capillary arteriovenous malformation; AVM, arteriovenous malformation.

Vascular malformations are a heterogeneous group of conditions commonly seen at our center and include simple and combined vascular malformations, such as simple lymphatic malformation, venous malformation, verrucous venous malformation (VVM), and combined capillary arteriovenous malformation (CAVM) (1). A VVM is a distinct venous malformation caused by a common venous malformation (2). It typically presents as a red or purple hyperkeratinized raised skin patch with a subcutaneous mass (2, 3). The subcutaneous mass is usually much more extensive than hyperkeratinized skin (4). Surgical excision is the first treatment of choice for VVM (2, 3, 5). Wide resection is essential to reduce the risk of postoperative recurrence (2, 5). Surgery of a lesion with a large subcutaneous lateral incision requires a wide and deep excision with a long linear incision. The treatment for CAVM has shifted to endovascular management (6). Ethanol embolization and sclerotherapy are currently considered as first-line treatments. However, endovascular measures cannot manage subcutaneous mass effects in the presence of fatty overgrowths. Surgical techniques play an important role in managing this condition (7–9) (Figure 1). Recently, we reported an endoscopic surgical technique for vascular anomalies, including lymphatic malformation, Klippel-Trénaunay syndrome, venous malformation, and intramuscular fibroadipose vascular anomaly (10). We have also used this technique to resect subcutaneous masses in VVM and CAVM. This study aimed to describe this innovative approach for VVM and CAVM, which involves both skin and subcutaneous tissues.

## 2 Materials and methods

### 2.1 Ethics approval

This study was approved by the Institutional Ethics Review Board of the Xi'an International Medical Center (approval number: XIMED-2024027. All patients gave consent for the publication of recognizable patient photographs or other identifiable materials, with the understanding that this information may be publicly available.

### 2.2 Patients

This retrospective study included patients with VVM and CAVM who underwent endoscopic resection underwent endoscopic resection for VVM or CAVM between September 2019 and July 2024 at Xi'an International Medical Center. The patients/parents were fully informed about the new technique and alternative treatments. Patients' data, including sex, age, diagnosis, symptoms, lesion site, previous interventions before surgery, surgery, and follow-up, were obtained from the Vascular Anomalies Center database between September 2019 and July 2024.

### 2.3 Diagnosis and treatment

Diagnosis was based on clinical and imaging evaluations by our multidisciplinary team, including pediatric interventional radiologists, an ultrasonologist, and pediatric surgeons. Imaging was used to confirm the diagnosis and detect the subcutaneous extension of the lesion. If the clinical characteristics were inconclusive, a biopsy was performed. Preoperative biopsy was not required in this cohort.

The diagnosis of classic VVM was based on following criteria: congenital red or purple nonkeratinized skin patches at the early age that slowly increased in size and color; blue subcutaneous mass and hyperkeratotic skin that gradually developed, often in childhood; ultrasonography demonstrating a subcutaneous solid mass with little venous flow signal; magnetic resonance imaging demonstrating subcutaneous high signals on T2-weighted fat-saturated sequences, typically consisting of nodules; specimens composed of blood-filled capillaries and small veins infiltrating the adipose tissue that are grossly and microscopically visible.

The subcutaneous variant (VVM-SV) is a specific form of VVM without obvious skin involvement (4). It spares the dermis and is confined to the subcutaneous layer (4). The history and typical clinical findings were similar to those of classic VVM, and compatible histopathological profiles were sufficient for a VVM-SV diagnosis (4).

Endoscopic surgery was indicated for obvious subcutaneous extension in the VVM and VVM-SV, fatty overgrowth in the CAVM, and failure of nonoperative therapy. Local resection of the dermal lesions of the VVM can be considered after endoscopic surgery. Ethanol-based endovascular therapy remains the first choice for patients with CAVM; however, some patients exhibit obvious fatty overgrowth that is not amenable to sclerotherapy. In these cases, surgery is recommended; however, it is often not curative. Management of residual CAVM through ethanol-based endovascular therapy (Figure 1), a microinjection technique, and a pulsed dye laser after endoscopic debulking surgery was planned. The microinjection technique for CM in CAVM has been reported previously. Endoscopic surgery was not considered in patients with excessive skin that required plastic surgery.

Endoscopic surgery is not radical for patients with VVMs. Further treatments for residual dermal patches include local resection, local flap transfer, and staged skin grafting. Surgery is typically radical for patients with VVM-SV.

### 2.4 Endoscopic surgery technique

Preoperative topographic markers were created on the skin to delineate the extent of the lesions based on imaging studies. The surgery was performed under endotracheal anesthesia. The patient was positioned to provide optimal exposure and access to the lesions. A tourniquet was routinely used to prevent bleeding, if possible. Initially, a 5-mm skin incision was made 5–10 cm away from the border of the marked area. A vascular tunneler was used to create and bluntly enlarge an initial working space. A 5-mm trocar was introduced into the subcutaneous layer through the initial incision. Two or three 3- and/or 5-mm trocars were usually placed in this manner. The VVM-SV is often a localized lesion. We preferred using 3-mm trocars to resect the lesion. Another trocar may be required during surgery to assist with the resection. After the placement of the trocar and creation of the initial workspace, CO_2_ gas was insufflated at a pressure of 6–12 mm Hg. A 0° or 30° endoscope was then placed via a port, and the subcutaneous nonvital neurovascular bundles were divided under endoscopic guidance using monopolar cautery or an ultrasonic scalpel. The skin was then elevated, and the working space was sufficiently enlarged. Pressure of 6–12 mm Hg was maintained during surgery. Resection was then started through the space between the skin and deep fascia. Silk traction sutures were placed for better exposure if required.

**Figure 2:**
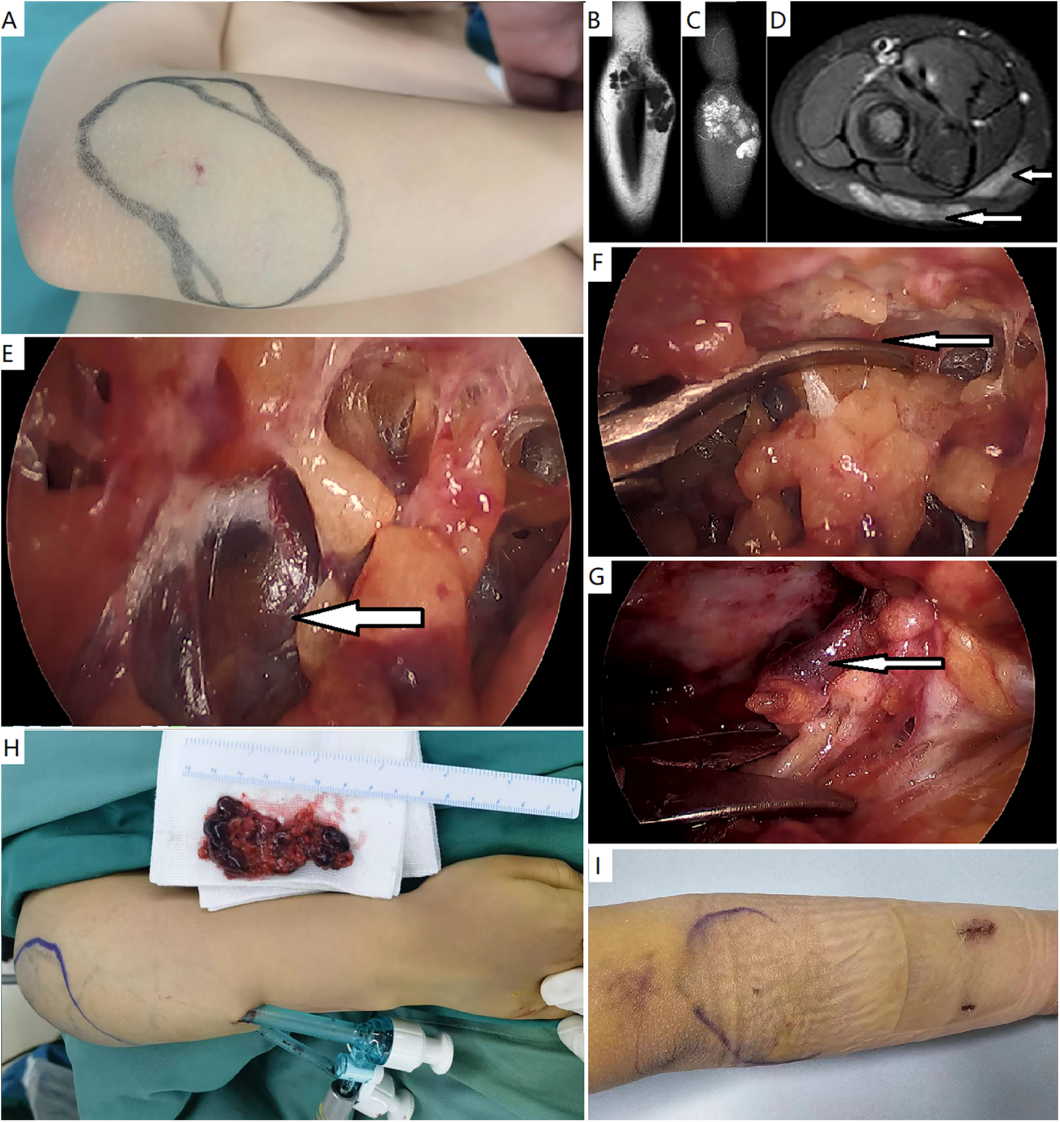
Endoscopic resection of a verrucous venous malformation—subcutaneous variant. **(A)** A female patient presented with a small skin patch and subcutaneous bluish hue in the right forearm. **(B**–**D)** Magnetic resonance imaging demonstrated a subcutaneous mass with high signals on T2-weighted fat-saturated sequences, consisting of nodules. Muscles were normal. **(E)** A subcutaneous workspace was created, and endoscopic resection was performed. The lesion appeared to be dark bluish nodules. **(F)** The lesion was divided initially just beneath the skin. **(G)** The lesion was elevated from the deep fascia. A definite margin was observed. **(H)** Gross specimen and the appearance of surgical site. **(I)** Appearance of the forearm after 3 days.

**Figure 3:**
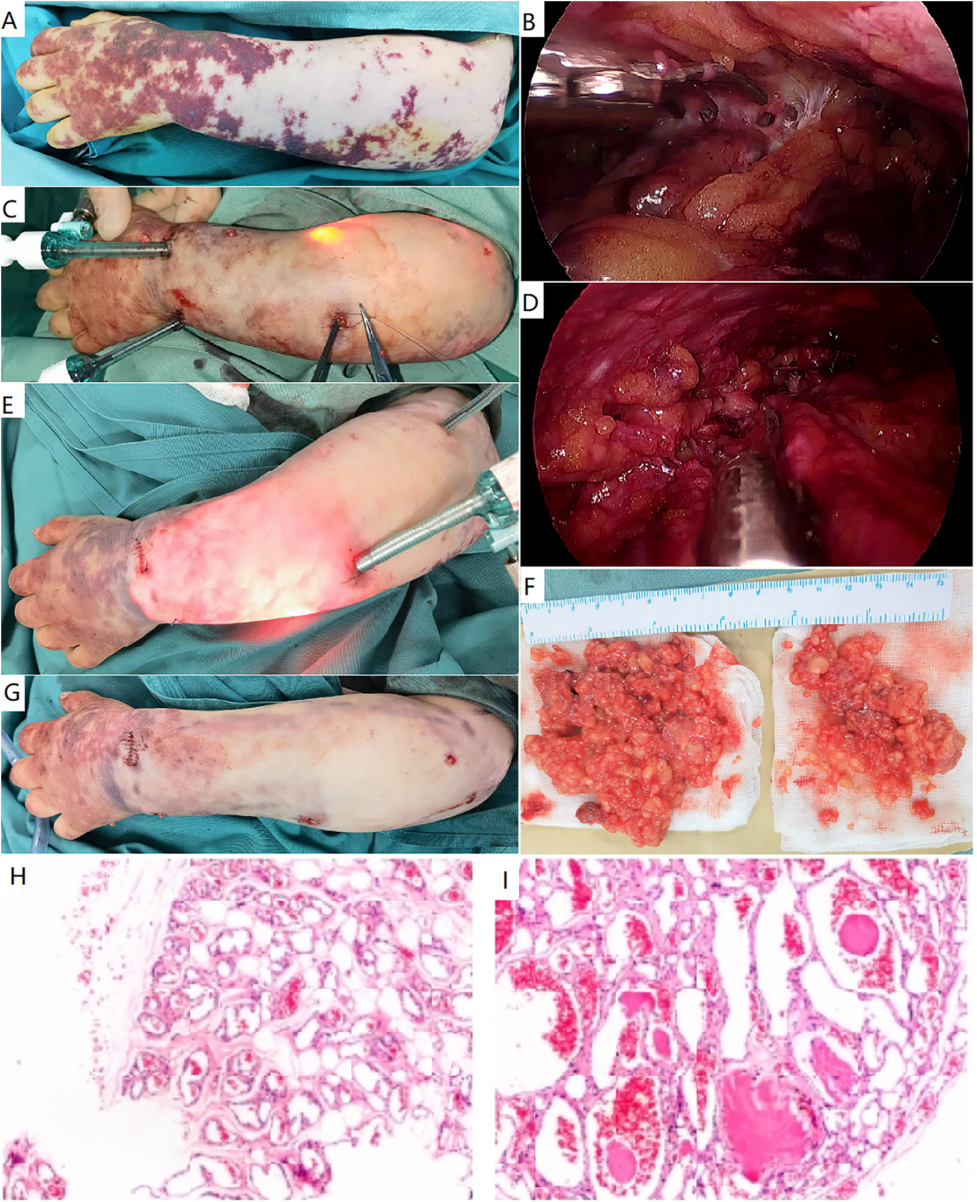
Endoscopic resection of a classic verrucous venous malformation. **(A)** A female patient presented with extensive hyperkeratotic skin patches and subcutaneous masses in her left arm and hand. A classic verrucous venous malformation was diagnosed. **(B–D)** Endoscopic resection of subcutaneous lesion. The lesion was initially divided beneath the dermis **(B)**, and additional ports were required **(C)**. The involved deep fascia was elevated and resected **(D)**. **(E)** More ports were placed to resect the extensive lesion. **(F)** Gross specimen of verrucous venous malformation, consisting of venous nodules. **(G)** Immediate appearance of the arm after surgery. **(H,I**) Histopathological study demonstrated adipose tissue was infiltrated by nodules of veins. These vessels ranged in size from capillaries to small veins. Thin fibrous and pericytic muscular walls can be seen in small and large channels.

**Figure 4:**
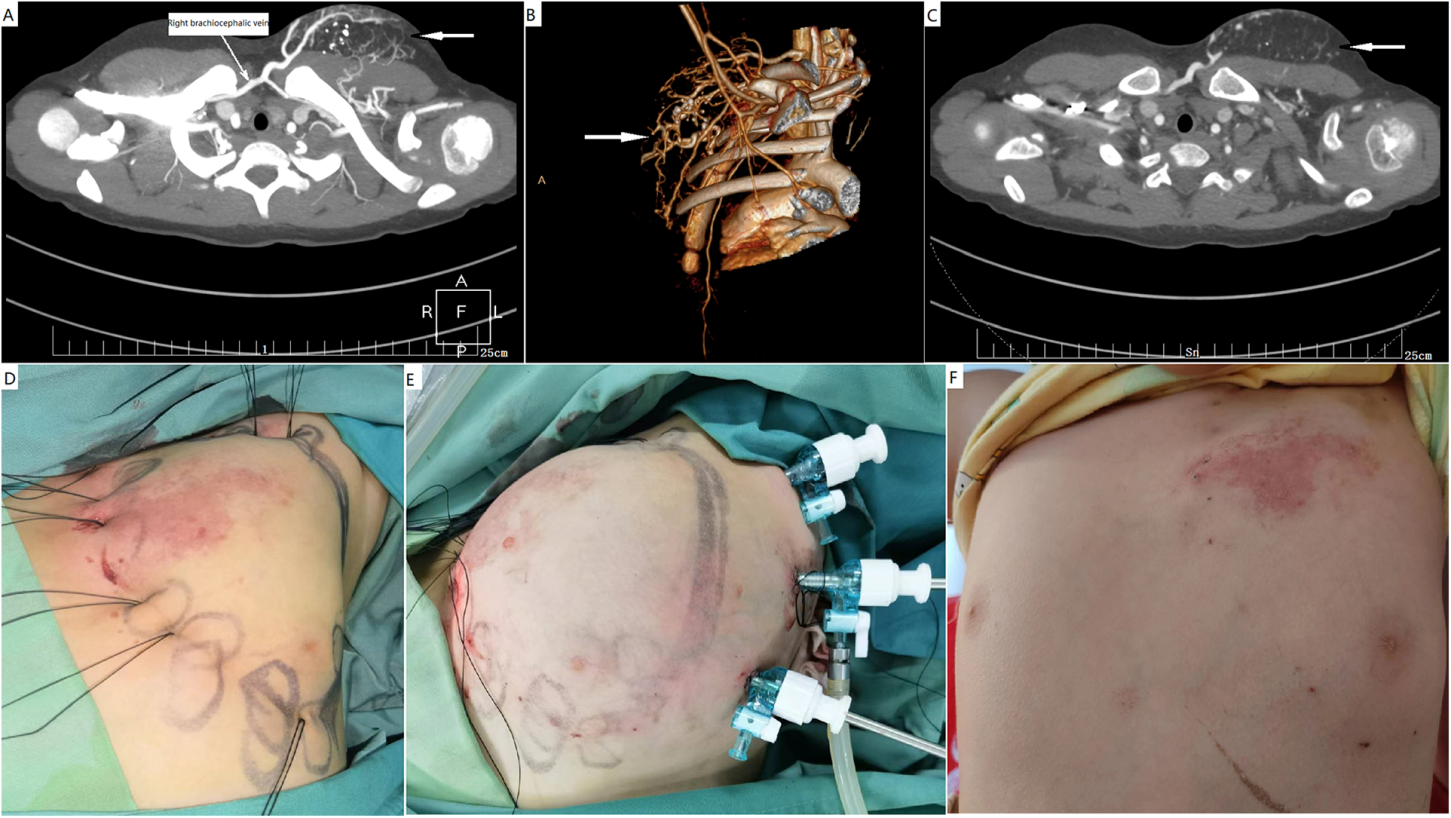
Endoscopic resection of a CAVM with fatty overgrowth in the left chest wall. **(A–C)** A male patient presented with skin capillary stain and a subcutaneous mass in the chest wall. Computed tomography studies with contrast enhancement and computed tomography angiography confirmed the diagnosis of CAVM. Vascular malformation was not the predominant component in the mass. **(D)** Preemptive percutaneous ligation of main feeding arteries and drainage veins was performed to secure bleeding. **(E)** The subcutaneous fatty overgrowth was endoscopically debulked. **(F)** Normal appearance of the surgical site after 3 months. CAVM, capillary malformation–arteriovenous malformation.

In patients with VVM and VVM-SV, the subcutaneous lesions, including the fat layer and deep fascia, were excised (Figure 2). A skin flap was created in a one-third or half of the circumference of the extremities in patients with extensive VVM. If the lesion involves more than half the circumference of the extremities, staged resection may be required to ensure skin flap survival (Figure 3). In patients with CAVM, as much overgrown adipose tissue as possible was resected until the expected local contour was obtained. The goal of the surgery was to improve the mass effect and appearance. Typically, radical resection is not possible. A tourniquet and preemptive percutaneous ligation of the main feeding arteries and drainage veins were used to prevent bleeding from the CAVM (Figure 4). Repeated intraoperative rinsing with saline was required to improve visibility, particularly in patients with extensive lesions or intraoperative bleeding.

After specimen retrieval, a suction drain was placed beneath the flap and removed via a port incision. Compressive elastic garments are routinely used for these purposes. The combination of suction drainage and compressive garments prevented blood collection under the flap, and circumferential compression allowed the flap to adhere to the underlying fascia and/or muscle. A drainage tube was not used in patients with VVM-SV.

## 3 Results

### 3.1 Clinical characteristics

The study's cohort included 14 female and 4 male patients, with ages ranging from 1 year to 8 years. Diagnoses included classic VVM (*n* = 10), VVM-SV (*n* = 4), and CAVM (*n* = 4). Surgical sites included the upper extremities (*n* = 9), lower extremities (*n* = 4), gluteal region (*n* = 3), and thoracic wall (*n* = 2) (Table 1). Patients with classic VVM typically presented with multiple hyperkeratotic purple patches and obvious subcutaneous bluish hue on a limb (Figure 3). In patients with VVM-SV, the skin patch was unremarkable, except for an underlying bluish mass (Figure 2A). All VVM-SVs were single and localized in the limbs. The lesion presented as a well-defined subcutaneous bluish nodule that was mobile and firmer than a common venous malformation (Figure 2). Patients with CAVM had multiple microarteriovenous fistulas associated with cutaneous capillary staining and excessive soft tissue growth at the affected location. The overgrown soft tissue predominantly contained solid, firm adipose, or fibroadipose components in the CAVM. Vascular malformations were not the main components. Therefore, intraoperative bleeding can be prevented by properly pre-ligating the main vessels or using a tourniquet. The dermal capillary stain of the CAVM and the skin patches of the VVM were not excised simultaneously.

**Table 1:** Patients’ clinical characteristics.

No	Sex	Age at surgery (years)	Diagnosis	Sites of lesion	Symptoms	Interventions before surgery	Surgery	Additional management after surgery
1	M	6	CAVM	Left chest wall	CAVM with adipose overgrowth	Transcatheter ethanol embolization	Debulking	Transcatheter ethanol embolization and microinjection of ethanol
2	F	6	CAVM	Left buttock	CAVM with adipose overgrowth	Transcatheter coils and ethanol embolization	Debulking	Transcatheter ethanol embolization and microinjection of ethanol
3	F	2	CAVM	Right buttock	CAVM with adipose overgrowth	None	Debulking	Transcatheter ethanol embolization
4	F	2	VVM	Left leg and ffoot	Hyperkeratotic skin patches and subcutaneous masses	Intralesional bleomycin injection	Radical resection of subcutaneous mass	None
5	M	1	VVM	Right thigh, leg and foot	Hyperkeratotic skin patches and subcutaneous masses; ulceration	Intralesional bleomycin injection	Partial resection of subcutaneous mass	None
6	F	6	VVM-SV	Right forearm	A small skin patch and subcutaneous mass	Intralesional bleomycin injection	Radical resection of subcutaneous mass	None
7	F	2	VVM	Right forearm	Hyperkeratotic skin patches and subcutaneous masses	None	Radical resection of subcutaneous mass	Skin patches resection
8	F	8	VVM	Left forearm and hand	Hyperkeratotic skin patches and subcutaneous masses	Oral sirolimus; intralesional bleomycin injection	Partial resection of subcutaneous mass	None
9	F	5	VVM	Left forearm and hand	Hyperkeratotic skin patches and subcutaneous masses	Oral sirolimus	Partial resection of subcutaneous mass	None
10	F	6	VVM	Left thigh, leg, and foot	Hyperkeratotic skin patches and subcutaneous masses; ulceration	Open partial excision; intralesional bleomycin injection	Partial resection of subcutaneous mass	None
11	F	2	VVM	Right leg	Hyperkeratotic skin patches and subcutaneous masses	None	Radical resection of subcutaneous mass	None
12	F	7	VVM-SV	Right hand	Small skin patches and subcutaneous mass	Intralesional bleomycin injection	Radical resection of subcutaneous mass	None
13	F	8	CAVM	Right buttock	CAVM with adipose overgrowth	None	Debulking	Transcatheter ethanol embolization
14	F	3	VVM	Right forearm	Hyperkeratotic skin patches and subcutaneous masses	Intralesional bleomycin injection	Radical resection of subcutaneous mass	None
15	F	3	VVM-SV	Left arm	Small skin patches and subcutaneous mass	Intralesional bleomycin injection	Radical resection of subcutaneous mass	None
16	M	2	VVM-SV	Left arm	Small skin patches and subcutaneous mass	None	Radical resection of subcutaneous mass	None
17	F	4	VVM	Left upper extremity and chest wall	Hyperkeratotic skin patches and subcutaneous masses	Open partial excision; intralesional bleomycin injection	Partial resection of subcutaneous mass	None
18	M	3	VVM	Left thigh, leg, and foot	Hyperkeratotic skin patches and subcutaneous masses; ulceration	Open partial excision; Intralesional bleomycin injection	Partial resection of subcutaneous mass	None

M, male; F, female; CAVM, capillary arteriovenous malformation; VVM, verrucous venous malformation; VVM-SV, verrucous venous malformation—subcutaneous variant.

### 3.2 Outcomes

The operative time ranged from 80 to 315 min (median, 180 min). Blood loss volume ranged from 5 to 50 ml. None of the patients received blood transfusions. The median follow-up duration after surgery was 13 months (range, 1–24 months). Curative resection was achieved in patients with VVM-SV. In patients with extensive VVM and CAVM, the extent of surgery is typically determined based on imaging studies. Surgery resulted in the radical resection of the subcutaneous part of the VVM and debulking of the overgrown adipose tissue in the CAVM. Technical success was achieved in all patients. Local skin necrosis (area <1 cm^2^) occurred in two patients, due to intra-operative local burn and/or tight bandaging. The drainage tube was successfully removed 7–10 days after surgery. No recurrence was observed during the short-term follow-up.

## 4 Discussion

According to the latest International Society for the Study of Vascular Anomalies classification, VVM and CAVM are categorized as vascular malformations, with the former being a low-flow malformation and the latter being a high-flow malformation (1). Both are congenital abnormal vascular channels that develop slowly. VVM was previously known as a verrucous hemangioma (1, 5). Currently, it is classified as a subtype of venous malformations that differs from common venous malformations (1). Surgical excision is the first-choice treatment for VVM, when feasible (2, 3). No recurrence is expected after resection if an adequate margin is obtained and the full depth of the lesion is removed (2, 5).

Open surgery has traditionally been the treatment of choice. Classic VVM usually manifest as relatively localized skin patches with extensive subcutaneous laterals on the limb. To reduce the risk of recurrence, a wide margin and full-depth lesion excision are required (2, 3, 5). Therefore, a long linear incision is often made during open surgery (3). A long linear scar is not aesthetically ideal. Additionally, a long scar on the joint may cause prolonged wound healing and excess scarring owing to constant joint motion.

Therefore, we performed endoscopic resection of the relatively extensive subcutaneous portion of the classic VVM. Multifocal skin patches were left for further dermal surgery to avoid long scars. A relatively extensive subcutaneous mass in the VVM can be successfully dissected and removed via endoscopic access. The VVM-SV can be completely resected using this minimally invasive approach. This approach can provide a relatively intact skin flap and preserve the completeness of the blood supply if properly elevated, especially in patients with extensive subcutaneous masses. More extensive resection than that of open surgery can be safely performed, even if the flap is thin.

In patients with CAVM with overgrowth, a combination of interventional radiological therapy and surgery is usually required (7, 9). Nonsurgical treatments include ethanol embolization, orally targeted therapy (trametinib) (11, 12), and laser therapy (13, 14). However, these treatments cannot successfully manage the subcutaneous part of the VVM and adipose overgrowth in CAVM. Debulking surgery is usually required in patients with obvious bulk or overgrowth. In these cases, the endoscopic approach has better cosmetic results than the traditional open surgery (15, 16).

Endoscopic resection of subcutaneous and intramuscular vascular anomalies has been reported previously (10). In the current cohort, the lesion included a superficial (skin) part, which has attendant cosmetic concerns. To achieve cosmetic improvement or cure, relatively extensive subcutaneous lesions must be removed. Surgery partially removed these lesions, which facilitated further intervention. Further management is aimed at treating local skin lesions or residual malformations. Our endoscopic approach cannot manage the superficial (skin) part of the lesion, except for superficial lymphatic malformations (10, 15).

This endoscopic surgical technique may provide valuable references for clinicians of other specialties, as it can be used for resecting other subcutaneous lesions and relatively extensive subcutaneous involvement in skin diseases. We did not perform a strict comparison between the endoscopic approach and traditional open surgery. However, it seems to markedly reduce the wound healing time and wound-related complications compared with open surgery at our center. This endoscopic surgical technique is a minimally invasive, feasible, and safe treatment for superficial vascular malformations. Furthermore, reduced surgical scarring and improved cosmetic results can be expected (16). The follow-up duration in this cohort was limited; therefore, appearance improvement and disease recurrence require constant monitoring.

Despite its benefits, soft tissue endoscopic surgery requires specialized instrument training and has a learning curve. Additionally, long-term data on certain procedures, such as endoscopic VVM resection, remain limited. Future research should focus on standardizing protocols and applications, and exploring applications in other anatomical regions, such as the neck and face. Furthermore, skin lesion, such as CM in the CAVM and skin patch of VVM, can't be managed by endoscopic approach.

In conclusion, endoscopic resection is a minimally invasive, feasible, and safe technique for VVM and in selected CAVM. This endoscopic surgical technique can achieve better cosmetic outcomes and reduce incision-related complications in patients with superficial lesions.

## Data Availability

The raw data supporting the conclusions of this article will be made available by the authors, without undue reservation.

## References

[1] International Society for the Study of Vascular Anomalies. ISSVA classification for vascular anomalies (2018). Available at: https://www.issva.org/UserFiles/file/ISSVA-Classification-2018.pdf (Accessed January 12, 2018).

[2] Wang H Guo Z. Localized verrucous venous malformations: treatment and response evaluation. J Am Acad Dermatol. (2022) 87:e105–6. 10.1016/j.jaad.2022.04.043 35504487

[3] Chang SJ Qiu Y Lin X. Authors’ reply: intralesional bleomycin injection for localized verrucous venous malformations. J Am Acad Dermatol. (2022) 87:e107–8. 10.1016/j.jaad.2022.04.044 35513178

[4] Schmidt BAR El Zein S Cuoto J Al-Ibraheemi A Liang MG Paltiel HJ Verrucous venous malformation-subcutaneous variant. Am J Dermatopathol. (2021) 43:e181–4. 10.1097/DAD.0000000000001963 33899768

[5] Calduch L Ortega C Navarro V Martínez E Molina I Jordá E. Verrucous hemangioma: report of two cases and review of the literature. Pediatr Dermatol. (2000) 17:213–7. 10.1046/j.1525-1470.2000.01755.x 10886755

[6] Müller-Wille R Wildgruber M Sadick M Wohlgemuth WA. Vascular anomalies (part II). Interventional therapy of peripheral vascular malformations. RoFo. (2018) 190(10):927–37. 10.1055/s-0044-101266 29415296

[7] Bayrak-Toydemir P Stevenson DA. Capillary malformation-arteriovenous malformation syndrome. In: Adam MP Mirzaa GM Pagon RA Wallace SE Amemiya A, editors. GeneReviews®. Seattle, WA: University of Washington (2011). p. 1–10. 21348050

[8] Zobel MJ Moses W Walther A Nowicki D Howell L Miller J Management challenges of a large upper extremity vascular malformation in a patient with capillary malformation-arteriovenous malformation syndrome. J Vasc Surg Venous Lymphat Disord. (2021) 9:781–4. 10.1016/j.jvsv.2020.07.001 32687897

[9] McCarthy C Deb S Maqusi S Gierman J. Giant chest wall arteriovenous malformation: a case report and literature review. Ann Vasc Surg. (2018) 46:369.e7–369.e11. 10.1016/j.avsg.2017.08.028 28890056

[10] Wang H Xie C Lin W Zhou J Yang W Guo Z. Endoscopic resection for vascular anomalies in children: a new standard. Ann Surg. (2023) 278:e870–5. 10.1097/SLA.0000000000005832 36825502 PMC10481932

[11] Al-Samkari H Eng W. A precision medicine approach to hereditary hemorrhagic telangiectasia and complex vascular anomalies. J Thromb Haemost. (2022) 20:1077–88. 10.1111/jth.15715 35343049 PMC10044495

[12] Nicholson CL Flanagan S Murati M Boull C McGough E Ameduri R Successful management of an arteriovenous malformation with trametinib in a patient with capillary-malformation arteriovenous malformation syndrome and cardiac compromise. Pediatr Dermatol. (2022) 39:316–9. 10.1111/pde.14912 35014097

[13] Iznardo H Roé E Puig L Vikula M López-Sánchez C Baselga E. Good response to pulsed dye laser in patients with capillary malformation-arteriovenous malformation syndrome (CM-AVM). Pediatr Dermatol. (2020) 37:342–4. 10.1111/pde.14095 31944370

[14] Ang YS Koh MJA. Treatment of capillary malformations in capillary malformation-arteriovenous malformation syndrome with pulsed dye laser. J Cosmet Dermatol. (2021) 20:3710–1. 10.1111/jocd.14042 33655684

[15] Wang H Guo X Liu Q Liu N He X Peng Y Liposuction-like sclerotherapy technique: a deep approach to superficial lymphatic malformations. J Am Acad Dermatol. (2019) 81:255–7. 10.1016/j.jaad.2019.01.082 30731174

[16] Xie C Wang H Guo Z Wang P Lin W Yang W. A novel endoscopic approach to fibroadipose vascular anomaly. J Pediatr Surg. (2025) 60:162064. 10.1016/j.jpedsurg.2024.162064 39616969

